# 2-(2-Methyl­naphtho[2,1-*b*]furan-1-yl)acetic acid

**DOI:** 10.1107/S1600536808015572

**Published:** 2008-05-30

**Authors:** Martyn Jevric, Dennis K. Taylor, Edward R. T. Tiekink

**Affiliations:** a Department of Chemistry, University of Adelaide, 5005 South Australia, Australia; b Department of Wine and Horticulture, University of Adelaide, Waite Campus, Glen Osmond 5064, South Australia, Australia; c Department of Chemistry, University of Texas at San Antonio, One UTSA Circle, San Antonio, Texas 78249-0698, USA

## Abstract

In the title mol­ecule, C_15_H_12_O_3_, the two six-membered and one five-membered fused-ring system is almost planar and the CH_2_C(=O)OH residue is essentially orthogonal to it. In the crystal structure, centrosymmetric dimers are formed *via* the carboxylic acid {⋯O=C—O—H}_2_ synthon.

## Related literature

For related literature, see: Haselgrove *et al.* (1999 ▶); Jevric *et al.* (2001 ▶).
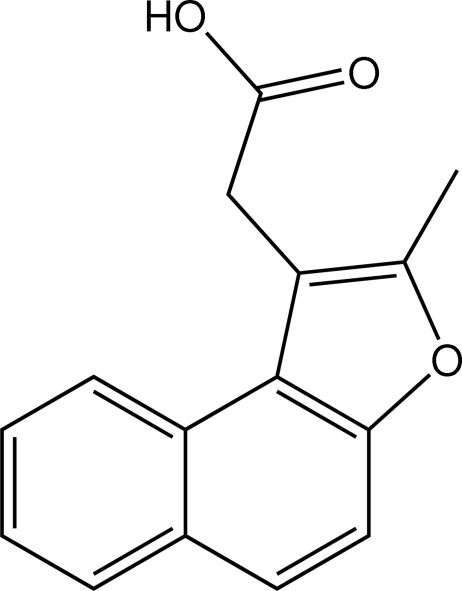


## Experimental

### 

#### Crystal data

C_15_H_12_O_3_
*M* *_r_* = 240.25
Monoclinic, 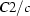

*a* = 31.380 (3) Å
*b* = 4.8370 (4) Å
*c* = 15.7885 (13) Å
β = 98.087 (2)°
*V* = 2372.6 (3) Å^3^
*Z* = 8
Mo *K*α radiation
μ = 0.09 mm^−1^
*T* = 223 (2) K
0.49 × 0.36 × 0.18 mm

#### Data collection

Bruker SMART CCD diffractometer
Absorption correction: none
9334 measured reflections
3445 independent reflections
2790 reflections with *I* > 2σ(*I*)
*R*_int_ = 0.026

#### Refinement

*R*[*F* ^2^ > 2σ(*F* ^2^)] = 0.047
*wR*(*F* ^2^) = 0.138
*S* = 1.04
3445 reflections
164 parameters
H-atom parameters constrained
Δρ_max_ = 0.34 e Å^−3^
Δρ_min_ = −0.15 e Å^−3^

### 

Data collection: *SMART* (Bruker, 2000 ▶); cell refinement: *SAINT* (Bruker, 2000 ▶); data reduction: *SAINT* and *SHELXTL* (Sheldrick, 2008 ▶); program(s) used to solve structure: *SIR92* (Altomare *et al.*, 1994 ▶); program(s) used to refine structure: *SHELXL97* (Sheldrick, 2008 ▶); molecular graphics: *ORTEPII* (Johnson, 1976 ▶) and *DIAMOND* (Brandenburg, 2006 ▶); software used to prepare material for publication: *SHELXL97*.

## Supplementary Material

Crystal structure: contains datablocks global, I. DOI: 10.1107/S1600536808015572/lh2631sup1.cif

Structure factors: contains datablocks I. DOI: 10.1107/S1600536808015572/lh2631Isup2.hkl

Additional supplementary materials:  crystallographic information; 3D view; checkCIF report

## Figures and Tables

**Table 1:** Hydrogen-bond geometry (Å, °)

*D*—H⋯*A*	*D*—H	H⋯*A*	*D*⋯*A*	*D*—H⋯*A*
O12—H12⋯O11^i^	0.83	1.83	2.6553 (14)	170
C21—H21*B*⋯O12^ii^	0.97	2.52	3.2663 (19)	134

Symmetry codes: (i) 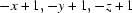; (ii) 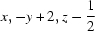.

**Fig. 1.**
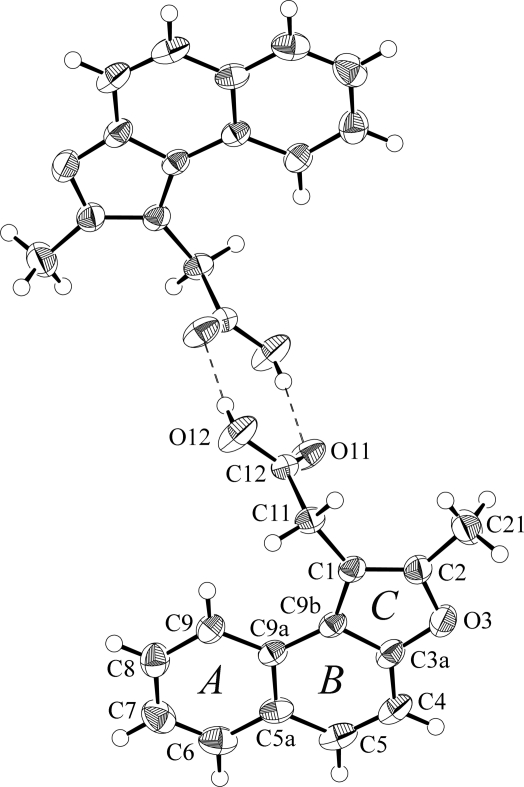
Molecular structure of (I) showing atom-labelling scheme and displacement ellipsoids at the 50% probability level. In addition an unlabelled symmetry related (1-x, 1-y, 1-z) molecule is shown to demonstrate the formation of a hydrogen bonded dimer.

**Fig. 2.**
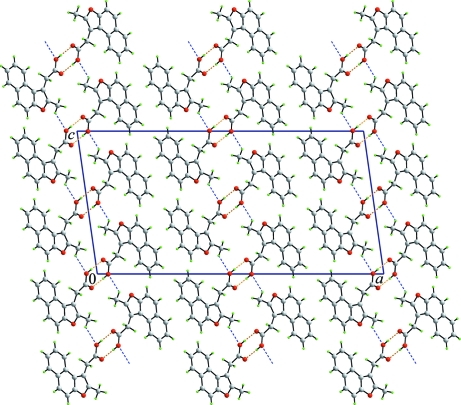
Crystal packing in (I) viewed in projection down the b axis highlighting the stacking of the 2-D arrays. Colour scheme red (O), grey (C), and green (H). The O—H···O (orange) and C—H···O (blue) contacts are shown as dashed lines.

**Fig. 3.**
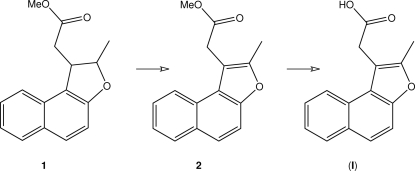
Reaction scheme.

## supplementary crystallographic information

### Comment

The effective dehydrogenation of diastereomeric mixture of 1, Fig. 3, to form the aromatized tricyclic 2 could be effected with the use of DDQ in THF under reflux conditions (Haselgrove *et al.*, 1999). Crystals of the title acid derivative (I) were obtained by base hydrolysis of 2 in methanol solution (Jevric *et al.*, 2001).

The tricyclic system in (I), Fig. 1, comprises six- (A), six- (B), and five-membered rings (C) with the sequence of dihedral angles between their respective least-squares planes being 1.48 (6), 2.34 (6), 0.91 (6) ° for A/B, A/C, and B/C, respectively. The CH_2_C(=O)OH residue is essentially orthogonal to this aromatic system as seen in the C1/C11/C12/O11 torsion angle of -165.68 (9)°. Centrosymmetrically related molecules associate into dimers *via* the familiar eight-membered carboxylic acid {···O=C—O—H}_2_ synthon, Table 1. Each dimer thus formed is associated to two other molecules, each related by 2-fold symmetry, *via* C—H···O contacts. These consolidate molecules into a 2-D array in the *bc*-plane as shown in Fig. 2.

### Experimental

To a stirring solution of 1 (Fig. 3, 1.91 g, 7.46 mmol) in anhydrous THF (50 ml) was added DDQ (1.81 g, 7.97 mmol) and the reaction brought to reflux for 24 h under a nitrogen atmosphere. The solution was allowed to cool to room temperature, diluted with water and extracted twice with dichloromethane. The organic phase was dried (MgSO_4_), filtered and volatiles removed in vacuo. The crude residue was purified by column chromatography (10% acetone in hexane) to give pure methyl 2-(2-methylnaphtho[2,1-*b*]furan-1-yl)acetate, 2, as a yellow solid, m.p: 349 - 351 K. *R*_f_ 0.41 (10% acetone in hexane). IR (CH_2_Cl_2_, cm^-1^) 1738, 1620, 1581, 1525, 804. ^1^H NMR (CDCl_3_, 300 MHz) δ 2.25 (s, 3H), 3.70 (s, 3H), 3.98 (s, 2H), 7.45–7.48 (m, 1H), 7.56–7.60 (m, 2H), 7.65–7.68 (m, 1H), 7.92–7.94 (m, 1H), 8.23–8.26 (m, 1H) p.p.m.. ^13^C NMR (CDCl_3_, 50 MHz) δ 12.0, 31.5, 52.2, 109.5, 112.1, 122.2, 122.8, 124.0, 124.6, 126.1, 128.0, 129.0, 130.8, 151.5, 152.2, 171.6 p.p.m.. MS m/*z* (%): 254 (*M*+, 76), 95 (100), 181 (31), 165 (27), 152 (22). HRMS, C_16_H_14_O_3_: calcd, 254.0943. Found 254.0942.

Compound (I) was obtained by the base hydrolysis of 2 in methanol solution. The colourless solid was recrystallized from ethanol solution in 75% yield; m.p.: 451 - 455 K (decomposes, sealed tube). IR (nujol, cm^-1^) 1699, 1622, 1579, 1525. ^1^H NMR (CDCl_3_, 300 MHz) δ 2.51 (s, 3H), 4.00 (s, 2H), 7.44–7.69 (m, 4H), 7.91–7.94 (m, 1H), 8.21–8.23 (m, 1H) p.p.m.. ^13^C NMR (CDCl_3_, 50 MHz) δ 11.9, 31.1, 108.8, 112.1, 122.0, 122.7, 124.0, 126.2, 127.9, 129.1, 130.8, 151.6, 152.4, 175.5 (1 masked carbon) p.p.m.. MS m/*z* (%): 240 (*M*+, 39), 195 (100), 165 (13), 152 (13), 69 (37). Elemental analysis found: C, 74.92; H, 4.98%. C_15_H_12_O_3_ requires C, 74.99; H, 5.03%.

### Refinement

All H atoms were included in the riding-model approximation, with C—H = 0.94 to 0.98 Å and O—H = 0.83 Å, and with *U*_iso_(H) = 1.5*U*_eq_(methyl-C and O) or 1.2*U*_eq_(remaining C).

### Crystal data

**Table:**

C_15_H_12_O_3_	*F*_000_ = 1008
*M**_r_* = 240.25	*D*_x_ = 1.345 Mg m^−^^3^
Monoclinic, *C*2/*c*	Mo *K*α radiation λ = 0.71069 Å
Hall symbol: -C 2yc	Cell parameters from 3301 reflections
*a* = 31.380 (3) Å	θ = 2.6–29.4º
*b* = 4.8370 (4) Å	µ = 0.09 mm^−^^1^
*c* = 15.7885 (13) Å	*T* = 223 (2) K
β = 98.087 (2)º	Block, colourless
*V* = 2372.6 (3) Å^3^	0.49 × 0.36 × 0.18 mm
*Z* = 8	

### Data collection

**Table:**

Bruker SMART CCD diffractometer	2790 reflections with *I* > 2σ(*I*)
Radiation source: fine-focus sealed tube	*R*_int_ = 0.026
Monochromator: graphite	θ_max_ = 30.0º
*T* = 223(2) K	θ_min_ = 2.6º
ω scans	*h* = −43→43
Absorption correction: none	*k* = −6→4
9334 measured reflections	*l* = −22→22
3445 independent reflections	

### Refinement

**Table:**

Refinement on *F*^2^	Secondary atom site location: difference Fourier map
Least-squares matrix: full	Hydrogen site location: inferred from neighbouring sites
*R*[*F*^2^ > 2σ(*F*^2^)] = 0.047	H-atom parameters constrained
*wR*(*F*^2^) = 0.138	*w* = 1/[σ^2^(*F*_o_^2^) + (0.0801*P*)^2^ + 0.5375*P*] where *P* = (*F*_o_^2^ + 2*F*_c_^2^)/3
*S* = 1.04	(Δ/σ)_max_ < 0.001
3445 reflections	Δρ_max_ = 0.34 e Å^−^^3^
164 parameters	Δρ_min_ = −0.15 e Å^−^^3^
Primary atom site location: structure-invariant direct methods	Extinction correction: none

### Special details

**Table:**

Geometry. All e.s.d.'s (except the e.s.d. in the dihedral angle between two l.s. planes) are estimated using the full covariance matrix. The cell e.s.d.'s are taken into account individually in the estimation of e.s.d.'s in distances, angles and torsion angles; correlations between e.s.d.'s in cell parameters are only used when they are defined by crystal symmetry. An approximate (isotropic) treatment of cell e.s.d.'s is used for estimating e.s.d.'s involving l.s. planes.
Refinement. Refinement of *F*^2^ against ALL reflections. The weighted *R*-factor *wR* and goodness of fit *S* are based on *F*^2^, conventional *R*-factors *R* are based on *F*, with *F* set to zero for negative *F*^2^. The threshold expression of *F*^2^ > σ(*F*^2^) is used only for calculating *R*-factors(gt) *etc*. and is not relevant to the choice of reflections for refinement. *R*-factors based on *F*^2^ are statistically about twice as large as those based on *F*, and *R*- factors based on ALL data will be even larger.

### Fractional atomic coordinates and isotropic or equivalent isotropic displacement parameters (Å^2^)

**Table:**

	*x*	*y*	*z*	*U*_iso_*/*U*_eq_	
O3	0.39156 (3)	0.5681 (2)	0.14890 (5)	0.0436 (2)	
O11	0.47261 (3)	0.47260 (18)	0.40193 (6)	0.0458 (2)	
O12	0.46215 (3)	0.7752 (2)	0.50211 (6)	0.0597 (3)	
H12	0.4808	0.6801	0.5312	0.090*	
C1	0.40206 (3)	0.6881 (2)	0.28846 (7)	0.0322 (2)	
C2	0.41414 (4)	0.7340 (3)	0.21078 (7)	0.0389 (3)	
C3a	0.36422 (4)	0.4133 (2)	0.19008 (7)	0.0371 (3)	
C4	0.33570 (4)	0.2173 (3)	0.14967 (8)	0.0445 (3)	
H4	0.3340	0.1805	0.0908	0.053*	
C5	0.31044 (4)	0.0821 (3)	0.19964 (8)	0.0447 (3)	
H5	0.2911	−0.0532	0.1748	0.054*	
C5a	0.31254 (3)	0.1401 (2)	0.28866 (8)	0.0374 (3)	
C6	0.28513 (4)	0.0037 (3)	0.33887 (10)	0.0480 (3)	
H6	0.2655	−0.1288	0.3131	0.058*	
C7	0.28646 (4)	0.0600 (3)	0.42402 (10)	0.0511 (3)	
H7	0.2675	−0.0308	0.4559	0.061*	
C8	0.31603 (4)	0.2532 (3)	0.46375 (8)	0.0451 (3)	
H8	0.3170	0.2900	0.5225	0.054*	
C9	0.34350 (4)	0.3890 (2)	0.41782 (7)	0.0360 (2)	
H9	0.3634	0.5163	0.4456	0.043*	
C9a	0.34237 (3)	0.3400 (2)	0.32913 (7)	0.0312 (2)	
C9b	0.36901 (3)	0.4774 (2)	0.27625 (7)	0.0309 (2)	
C11	0.42022 (3)	0.8343 (2)	0.36877 (7)	0.0336 (2)	
H11A	0.3967	0.8781	0.4012	0.040*	
H11B	0.4329	1.0095	0.3535	0.040*	
C12	0.45400 (3)	0.6725 (2)	0.42568 (6)	0.0306 (2)	
C21	0.44569 (4)	0.9241 (4)	0.17989 (10)	0.0560 (4)	
H21A	0.4541	1.0645	0.2229	0.084*	
H21B	0.4327	1.0118	0.1273	0.084*	
H21C	0.4709	0.8204	0.1693	0.084*	

### Atomic displacement parameters (Å^2^)

**Table:**

	*U*^11^	*U*^22^	*U*^33^	*U*^12^	*U*^13^	*U*^23^
O3	0.0459 (5)	0.0549 (5)	0.0297 (4)	0.0104 (4)	0.0047 (3)	0.0020 (3)
O11	0.0497 (5)	0.0434 (5)	0.0394 (5)	0.0167 (4)	−0.0108 (4)	−0.0094 (3)
O12	0.0662 (6)	0.0695 (7)	0.0373 (5)	0.0348 (5)	−0.0140 (4)	−0.0176 (4)
C1	0.0310 (5)	0.0332 (5)	0.0311 (5)	0.0067 (4)	0.0001 (4)	0.0038 (4)
C2	0.0352 (5)	0.0463 (6)	0.0347 (5)	0.0086 (4)	0.0029 (4)	0.0069 (5)
C3a	0.0392 (5)	0.0411 (6)	0.0293 (5)	0.0108 (4)	−0.0013 (4)	−0.0013 (4)
C4	0.0490 (6)	0.0471 (7)	0.0334 (6)	0.0121 (5)	−0.0089 (5)	−0.0100 (5)
C5	0.0424 (6)	0.0386 (6)	0.0476 (7)	0.0056 (5)	−0.0129 (5)	−0.0106 (5)
C5a	0.0329 (5)	0.0316 (5)	0.0447 (6)	0.0052 (4)	−0.0055 (4)	−0.0004 (4)
C6	0.0395 (6)	0.0379 (6)	0.0636 (8)	−0.0032 (5)	−0.0037 (5)	0.0045 (6)
C7	0.0460 (7)	0.0459 (7)	0.0624 (9)	−0.0033 (5)	0.0110 (6)	0.0150 (6)
C8	0.0519 (7)	0.0433 (6)	0.0407 (6)	0.0039 (5)	0.0087 (5)	0.0085 (5)
C9	0.0405 (5)	0.0331 (5)	0.0335 (5)	0.0031 (4)	0.0019 (4)	0.0016 (4)
C9a	0.0312 (5)	0.0281 (5)	0.0325 (5)	0.0070 (4)	−0.0016 (4)	0.0004 (4)
C9b	0.0318 (5)	0.0306 (5)	0.0284 (5)	0.0075 (4)	−0.0017 (4)	−0.0010 (4)
C11	0.0338 (5)	0.0294 (5)	0.0357 (5)	0.0029 (4)	−0.0013 (4)	0.0016 (4)
C12	0.0289 (4)	0.0312 (5)	0.0305 (5)	−0.0006 (4)	0.0005 (3)	−0.0001 (4)
C21	0.0455 (7)	0.0702 (10)	0.0545 (8)	0.0040 (6)	0.0150 (6)	0.0185 (7)

### Geometric parameters (Å, °)

**Table:**

O3—C3a	1.3697 (15)	C5a—C9a	1.4323 (15)
O3—C2	1.3807 (15)	C6—C7	1.366 (2)
O11—C12	1.2157 (13)	C6—H6	0.9400
O12—C12	1.2968 (13)	C7—C8	1.401 (2)
O12—H12	0.8300	C7—H7	0.9400
C1—C2	1.3519 (16)	C8—C9	1.3697 (17)
C1—C9b	1.4474 (15)	C8—H8	0.9400
C1—C11	1.4930 (15)	C9—C9a	1.4157 (15)
C2—C21	1.4832 (18)	C9—H9	0.9400
C3a—C9b	1.3829 (15)	C9a—C9b	1.4261 (15)
C3a—C4	1.3950 (17)	C11—C12	1.5084 (14)
C4—C5	1.362 (2)	C11—H11A	0.9800
C4—H4	0.9400	C11—H11B	0.9800
C5—C5a	1.4255 (18)	C21—H21A	0.9700
C5—H5	0.9400	C21—H21B	0.9700
C5a—C6	1.4127 (18)	C21—H21C	0.9700
			
C3a—O3—C2	105.97 (9)	C9—C8—H8	119.7
C12—O12—H12	109.5	C7—C8—H8	119.7
C2—C1—C9b	106.37 (10)	C8—C9—C9a	120.89 (11)
C2—C1—C11	124.78 (11)	C8—C9—H9	119.6
C9b—C1—C11	128.84 (10)	C9a—C9—H9	119.6
C1—C2—O3	111.41 (11)	C9—C9a—C9b	124.47 (10)
C1—C2—C21	133.27 (12)	C9—C9a—C5a	118.55 (11)
O3—C2—C21	115.32 (11)	C9b—C9a—C5a	116.98 (10)
O3—C3a—C9b	110.84 (10)	C3a—C9b—C9a	118.67 (10)
O3—C3a—C4	123.97 (10)	C3a—C9b—C1	105.40 (10)
C9b—C3a—C4	125.19 (11)	C9a—C9b—C1	135.91 (9)
C5—C4—C3a	116.72 (11)	C1—C11—C12	114.36 (9)
C5—C4—H4	121.6	C1—C11—H11A	108.7
C3a—C4—H4	121.6	C12—C11—H11A	108.7
C4—C5—C5a	121.79 (11)	C1—C11—H11B	108.7
C4—C5—H5	119.1	C12—C11—H11B	108.7
C5a—C5—H5	119.1	H11A—C11—H11B	107.6
C6—C5a—C5	120.93 (11)	O11—C12—O12	123.46 (9)
C6—C5a—C9a	118.45 (11)	O11—C12—C11	123.82 (9)
C5—C5a—C9a	120.62 (11)	O12—C12—C11	112.68 (9)
C7—C6—C5a	121.48 (12)	C2—C21—H21A	109.5
C7—C6—H6	119.3	C2—C21—H21B	109.5
C5a—C6—H6	119.3	H21A—C21—H21B	109.5
C6—C7—C8	120.00 (12)	C2—C21—H21C	109.5
C6—C7—H7	120.0	H21A—C21—H21C	109.5
C8—C7—H7	120.0	H21B—C21—H21C	109.5
C9—C8—C7	120.60 (12)		

### Hydrogen-bond geometry (Å, °)

**Table:**

*D*—H···*A*	*D*—H	H···*A*	*D*···*A*	*D*—H···*A*
O12—H12···O11^i^	0.83	1.83	2.6553 (14)	170
C21—H21B···O12^ii^	0.97	2.52	3.2663 (19)	134

Symmetry codes: (i) −*x*+1, −*y*+1, −*z*+1; (ii) *x*, −*y*+2, *z*−1/2.

## References

[bb1] Altomare, A., Cascarano, M., Giacovazzo, C., Guagliardi, A., Burla, M. C., Polidori, G. & Camalli, M. (1994). *J. Appl. Cryst.* **27**, 435–435.

[bb2] Brandenburg, K. (2006). *DIAMOND* Crystal Impact GbR, Bonn, Germany.

[bb3] Bruker (2000). *SMART* and *SAINT* Bruker AXS Inc., Madison, Wisconsin, USA.

[bb4] Haselgrove, T. D., Jevric, M., Taylor, D. K. & Tiekink, E. R. T. (1999). *Tetrahedron*, **55**, 14739–14762.

[bb5] Jevric, M., Taylor, D. K. & Tiekink, E. R. T. (2001). *Z. Kristallogr.* **216**, 543–544.

[bb6] Johnson, C. K. (1976). *ORTEPII* Report ORNL-5138. Oak Ridge National Laboratory, Tennessee, USA.

[bb7] Sheldrick, G. M. (2008). *Acta Cryst.* A**64**, 112–122. 10.1107/S0108767307043930 18156677

